# Third generation EGFR inhibitor osimertinib combined with pemetrexed or cisplatin exerts long-lasting anti-tumor effect in EGFR-mutated pre-clinical models of NSCLC

**DOI:** 10.1186/s13046-019-1240-x

**Published:** 2019-05-28

**Authors:** Silvia La Monica, Roberta Minari, Daniele Cretella, Lisa Flammini, Claudia Fumarola, Mara Bonelli, Andrea Cavazzoni, Graziana Digiacomo, Maricla Galetti, Denise Madeddu, Angela Falco, Costanza Annamaria Lagrasta, Anna Squadrilli, Elisabetta Barocelli, Alessandro Romanel, Federico Quaini, Pier Giorgio Petronini, Marcello Tiseo, Roberta Alfieri

**Affiliations:** 1 Department of Medicine and Surgery, University of Parma, Parma, Italy; 2 Medical Oncology Unit, University Hospital of Parma, Parma, Italy; 3 Food and Drug Department, University of Parma, Parma, Italy; 4 Italian Workers’ Compensation Authority (INAIL) Research Center, Parma, Italy; 5 Center of Excellence for Toxicological Research (CERT), University of Parma, Parma, Italy; 6 Centre for Integrative Biology (CIBIO), University of Trento, Trento, Italy

**Keywords:** Non-Small Cell Lung Cancer, Osimertinib, Resistance, Epidermal Growth Factor Receptor, Pemetrexed, Cisplatin

## Abstract

**Background:**

The third generation Epidermal Growth Factor Receptor (EGFR) Tyrosine Kinase Inhibitor (TKI) osimertinib has been initially approved for T790M positive Non-Small Cell Lung Cancer (NSCLC) and more recently for first-line treatment of *EGFR*-mutant T790M negative NSCLC patients. Similarly to previous generation TKIs, despite the high response rate, disease progression eventually occurs and current clinical research is focused on novel strategies to delay the emergence of osimertinib resistance. In this study we investigated the combination of osimertinib with pemetrexed or cisplatin in EGFR-mutated NSCLC cell lines and xenografts.

**Methods:**

Tumor growth was evaluated in a PC9T790M xenograft model and tissue composition was morphometrically determined. PC9, PC9T790M and HCC827 cell lines were employed to test the efficacy of osimertinib and chemotherapy combination in vitro. Cell viability and cell death were evaluated by MTT assay and fluorescence microscopy. Protein expression and gene status were analysed by Western blotting, fluorescence in situ hybridization analysis, next-generation sequencing and digital droplet PCR.

**Results:**

In xenograft models, osimertinib significantly inhibited tumor growth, however, as expected, in 50% of mice drug-resistance developed. A combination of osimertinib with pemetrexed or cisplatin prevented or at least delayed the onset of resistance. Interestingly, such combinations increased the fraction of fibrotic tissue and exerted a long-lasting activity after stopping therapy*.* In vitro studies demonstrated the stronger efficacy of the combination over the single treatments in inhibiting cell proliferation and inducing cell death in PC9T790M cells as well as in T790M negative PC9 and HCC827 cell lines, suggesting the potential role of this strategy also as first-line treatment. Finally, we demonstrated that osimertinib resistant clones, either derived from resistant tumors or generated in vitro, were less sensitive to pemetrexed prompting to use a chemotherapy regimen non-containing pemetrexed in patients after progression to osimertinib treatment.

**Conclusions:**

Our results identify a combination between osimertinib and pemetrexed or cisplatin potentially useful in the treatment of EGFR-mutated NSCLC patients, which might delay the appearance of osimertinib resistance with long-lasting effects.

**Electronic supplementary material:**

The online version of this article (10.1186/s13046-019-1240-x) contains supplementary material, which is available to authorized users.

## Background

Epidermal Growth Factor Receptor (*EGFR*) mutation-positive lung cancer occurs in about 10–15% of Caucasian patients and 40–50% of Japanese patients. Non-Small Cell Lung Cancer (NSCLC) patients harboring *EGFR* activating mutations, such as in-frame deletions in exon 19 (Ex19del) or missense mutation in exon 21(L858R), show high sensitivity to EGFR tyrosine kinase inhibitors (TKIs) such as gefitinib, erlotinib, afatinib and dacomitinib. However, the acquisition of *EGFR* T790M secondary mutation, the substitution of threonine 790 with methionine, is responsible for half of the cases of acquired resistance to TKI treatment [1].

Osimertinib, a third-generation EGFR-TKI that selectively binds the C797 residue inhibiting the T790M mutation, has shown high activity in term of Progression-Free Survival (PFS) and overall response rate in *EGFR*-T790M positive patients [2, 3] and efficacy superior to gefitinib/erlotinib in the first-line treatment by approximately a 9 months advantage in PFS [4]. However, acquired resistance occurs also to osimertinib either in T790M-positive NSCLC patients or in patients treated in first-line [5, 6]. EGFR-dependent or -independent mechanisms of resistance have been described even if they remain not completely understood [5]. *EGFR* G796/C797, L792 and L718/G719 mutations, *MET* and *HER2* amplification, *BRAF*, *KRAS*, and *PIK3CA* mutations, oncogenic fusion mutations in *FGFR3*, *RET*, and *NTRK* were recently identified in a large cohorts of osimertinib-resistant lung cancer patients either treated in second-line [7, 8] and in first-line [9]. Knowledge of these mechanisms is relevant in order to develop new therapeutic strategies to overcome TKI-resistance; however, how prevent or delay the acquisition of resistance remains an important issue. Our previous data indicated that in PC9 cell line and xenograft models the combination of gefitinib with pemetrexed or the intermittent combination of pemetrexed and gefitinib prevented the appearance of gefitinib resistance mediated by T790M mutation and epithelial-mesenchymal transition [10]; however, the combination was ineffective when gefitinib was administered before pemetrexed. Theoretically, chemotherapy, given its different and more generic mechanism of action, can postpone the resistance to EGFR-TKIs by limiting the tumor heterogeneity, thus improving the efficacy of treatment either in first- and second-line.

In view of this recent very favorable experience, osimertinib combined or intercalated with chemotherapy deserves to be considered either for patients in progression after first/second-generation TKIs or in first-line setting. Neither preclinical nor clinical data are available to date.

Pemetrexed-platinum based chemotherapy remains the standard of care for T790M negative patients progressing after first-line EGFR-TKIs and for patients with T790M positive tumors in progression after second-line osimertinib [11].

Our study was undertaken to explore the combination of osimertinib with pemetrexed or cisplatin in vivo in a mouse model of PC9T790M xenograft and in vitro in PC9T790M, PC9 and HCC827 cell lines.

## Methods

### Cell lines and culture

The NSCLC cell line PC9, harboring an in-frame deletion in exon 19 of *EGFR* gene, was kindly provided by Dr. P. Jänne (Dana-Farber Cancer Institute, Boston MA). PC9T790M cell clone was generated by exposing PC9 parental cells to increasing concentrations of gefitinib [10]. This cell clone was cultured in the presence of gefitinib 1 μM to maintain a selection pressure during in vitro propagation. HCC827 cell line was from ATCC (Manassas, VA). PC9T790M clones resistant to osimertinib were isolated after 9 months of culturing PC9T790M cells in the presence of increasing concentrations of osimertinib (from 10 nM to 500 nM). Clones were cultured in the presence of 500 nM osimertinib. Cells were cultured in RPMI-1640 (Life Technologies, Gaithersburg, MD) medium supplemented with 10% fetal bovine serum (Life Technologies) and maintained under standard cell culture conditions at 37 °C in a water-saturated atmosphere of 5% CO_2_ in air.

### Drug treatment

Osimertinib was provided by AstraZeneca (Milan, Italy). Pemetrexed and cisplatin were from inpatient pharmacy of University Hospital of Parma. Osimertinib was dissolved in DMSO (Sigma, ST Louis, MO), while pemetrexed and cisplatin were dissolved in 0.9% sodium chloride solution and diluted in fresh medium before use. Final DMSO concentration in medium never exceeded 0.1% (v/v) and equal amounts of the solvent were added to control cells.

### Analysis of cell proliferation, cell viability and cell death

Cell number, cell viability and cell death were evaluated as previously described [12].

### Western blot analysis

Procedures for protein extraction, solubilization, and protein analysis by 1-D PAGE are described elsewhere [13]. Antibody against Thymidylate Synthase (TS) was from Upstate (Lake Placid, NY); antibody against MET, p-EGFRTyr1068, EGFR, pAKTser473, AKT, p-ERK1/2Thr202/Tyr204, ERK1/2, Bim, cleaved caspase-7, PARP and HRP-conjugated secondary antibodies was from Cell Signaling Technology (Beverly, MA); antibody against Actin was from Sigma; and chemoluminescence system (ImmobilionTM Western Chemiluminescent HRP Substrate) was from Millipore (Temecula, CA). Reagents for electrophoresis and blotting analysis were from BIO-RAD (Hercules, CA).

### Tumor xenografts

A total of 5 × 10^6^ PC9T790M cells were suspended in 200 μL of Matrigel (BD Biosciences, Erembodegem, Belgium)/PBS (1:1) and were subcutaneously injected in the flank of Balb/c-Nude female mice (Charles River Laboratories, Calco, Italy). The animals were housed in a protected unit for immunodeficient animals with 12-h light-dark cycles and provided with sterilized food and water ad libitum. When tumor volume reached an average size of 150mm^3^, animals (*N* = 8) were treated with osimertinib (3 mg/kg in 1% Tween 80) given once per day, five times per week, by oral gavage; pemetrexed (100 mg/kg in 0.9% NaCl) [10], cisplatin (4 mg/kg in 0.9% NaCl) [14] or vehicle alone (control group) were administered intraperitoneally once a day, twice per week. Tumor xenografts were measured as previously described [15]. At the end of the experiments, mice were euthanized by cervical dislocation and tumors weighted and collected for immunohistochemical and next-generation sequencing (NGS) analysis.

All experiments involving animals and their care were performed with the approval of the Local Ethical Committee of University of Parma (Organismo per la Protezione e il Benessere degli Animali) and by the Italian Ministry of Health, in accordance with the institutional guidelines that are in compliance with national (D.Lgs. 26/2014) and international (Directive 2010/63/EU) laws and policies.

### Isolation and in vitro expansion of neoplastic cells from xenograft tumors

Tissues from osimertinib-resistant tumors were enzymatically digested using the Tumor Dissociation Kit from Miltenyi Biotec (Bergisch Gladbach, Germany) and the gentleMACS™ Dissociator (Miltenyi Biotec) was used for the mechanical dissociation. The single-cell suspension obtained after digestion was enriched in tumor cells using the Tumor Cell Isolation Kit (Miltenyi Biotec) accordingly to manufacturer’s instructions, as previously described [10]. Tumor cells were cultured with 500 nM osimertinib to maintain a selection pressure during in vitro propagation.

### Morphometric analysis of tumor xenografts

Subcutaneous nodules were excised, formalin fixed, paraffin embedded and processed for histochemical analysis. The morphometric evaluation of xenograft composition was performed on Masson’s trichrome-stained sections. In detail, the number of points overlying neoplastic tissue, fibrosis or necrosis was counted and expressed as percentage of the total number of points explored to define the volume fractions of each tissue component. All these morphometric measurements were obtained with the aid of a grid defining a tissue area of 0.22 mm^2^ and containing 42 sampling points each covering an area of 0.0052 mm^2^. These evaluations were performed on the entire section of each tumor sample using an optical microscope (200X final magnification).

### Fluorescence in situ hybridization (FISH) analysis

FISH analysis of *EGFR*, *MET*, and *HER2* genes was assessed on histologic samples to evaluate their amplification within the tumor xenografts. Briefly, following DNA denaturation, specific fluorescent probes, complementary to the analyzed genes and to the centromeric region of the chromosome (CEP), were applied and sections were post-hybridized according to manufacturer’s instructions. Nuclei were counterstained with DAPI, and the images were acquired and analyzed at 1000X magnification using a fluorescence microscope (Nikon) with Z-stack equipment. Tumors were defined amplified if EGFR/CEP7, MET/CEP7, HER2/CEP17 ratio was ⩾2.

### Next-generation sequencing and digital droplet PCR

NGS was performed with Solid Tumor SolutionTM (Sophia Genetics) on the MiSeq platform (Illumina®, San Diego, CA). Genomic DNA was extracted from tumor nodules with Qiagen DNA Mini Kit (Qiagen®, Valencia, CA, USA) and quantified with QuantiFluor® dsDNA System (Promega). Each libraries were prepared starting from 65 ng of genomic DNA, in according to manufacturer’s protocol. 10pM of diluted and denatured libraries were loaded on MiSeq Reagent Kit v3. Data were analyzed with Sophia DDM platform (Sophia Genetics). This NGS panel allowed to provide a comprehensive assessment of Single Nucleotide Variation (SNV) and Indels in hotspot of 42 genes (*AKT1, ALK, BRAF, CDK4, CDKN2A, CTNNB1, DDR2, DICER1, EGFR, ERBB2, ERBB4, FBXW7, FGFR1, FGFR2, FGFR3, FOXL2, GNA11, GNAQ, GNAS, H3F3A, H3F3B, HIST1H3B, HRAS, IDH1 IDH2, KIT, KRAS, MAP 2 K1, MET, MYOD1, NRAS, PDGFRA, PIK3CA, PTPN11, RAC1, RAF1, RET, ROS1, SF3B1, SMAD4, TERT,TP53*) and gene amplification events over 24 genes which are *ALK, BRAF, CDK4, CDKN2A, EGFR, ERBB2, FBXW7, FGFR1, FGFR2, FGFR3, HRAS, KIT, KRAS, MET, MYOD1, NRAS, PDGFRA, PIK3CA, RAF1, ROS1, RET, SF3B1, TERT* and TP53.

Digital droplet PCR (ddPCR) was performed on tumor DNA to assess the presence of tertiary *EGFR* mutation. Site specific mutation detection assays were performed to study *EGFR* C797, L792 and L718 mutations and copy number assays were performed to assess the amplification of *EGFR* and *NRAS* as a potential mechanism of resistance.

### Statistical analysis

Statistical analyses were carried out using GraphPad Prism version 6.0 software (GraphPad Software Inc., San Diego, CA). Results are expressed as mean values ±standard deviations (SD) for the indicated number of independent measurements. Differences between the mean values recorded for different experimental conditions were evaluated by Student’s t-test or by one-way ANOVA followed by Bonferroni’s post-test, and *p* values are indicated where appropriate in the figures and in their legends. *P* values < 0.05 were considered as significant. For in vivo studies comparison among groups was made using two-way repeated measures ANOVA followed by Bonferroni’s post-test (to adjust for multiple comparisons). Adjusted *p* values of less than 0.05 were considered significant.

## Results

### Efficacy of osimertinib combined with pemetrexed or cisplatin on tumor growth in PC9T790M xenograft and cell line models

Firstly, the effects of the combination of osimertinib with pemetrexed were investigated on PC9T790M (*EGFR* exon 19 E746-A750 deletion, T790M positive) xenograft models.

**Fig. 1:**
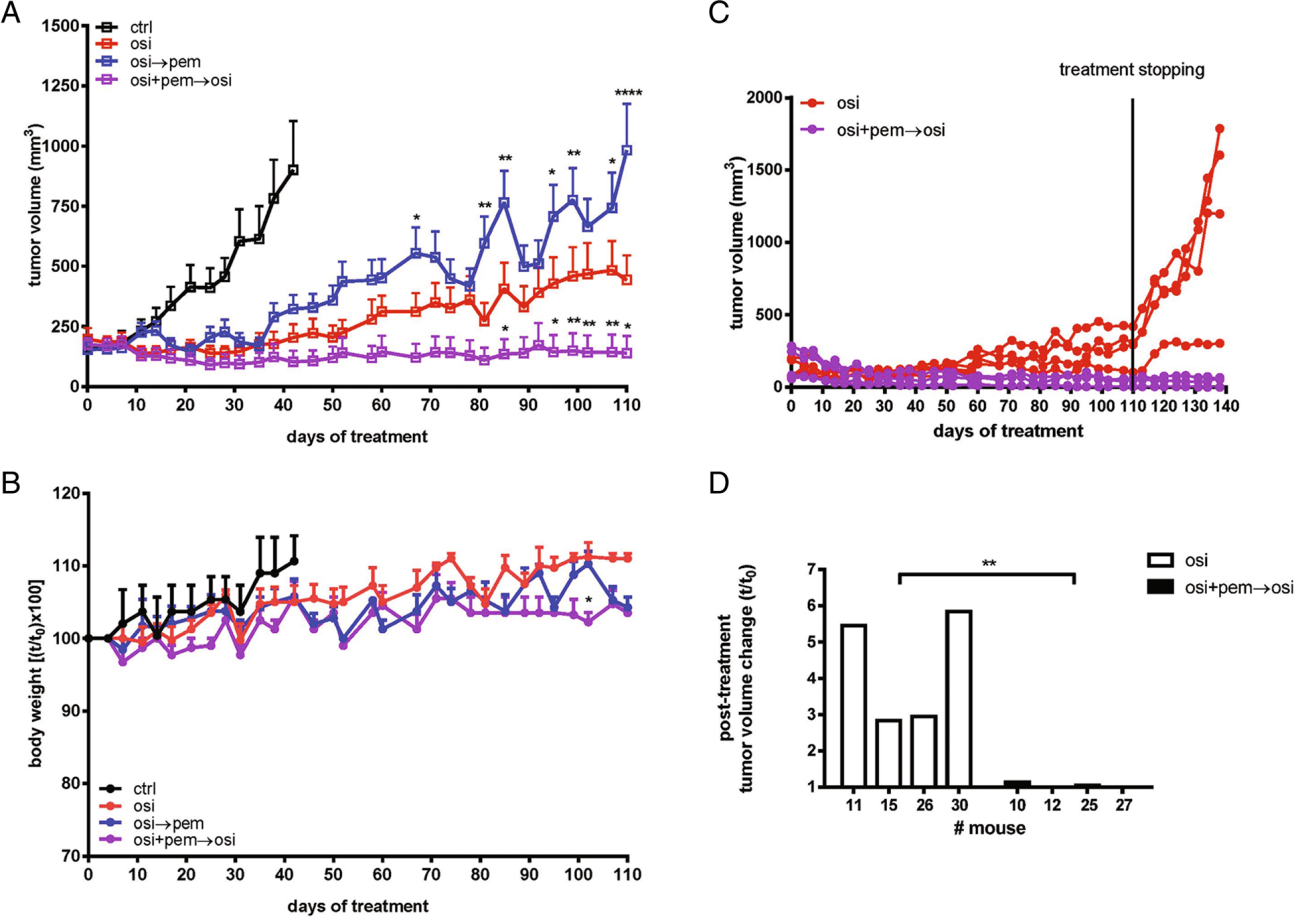
Effects of osimertinib and pemetrexed association in PC9T790M xenograft model. **a** PC9T790M cells were subcutaneously inoculated into BALB/C nude female mice, and after tumors had reached an average size of approximately 150mm^3^ the animal were treated with vehicle alone (ctrl), osimertinib (3 mg/kg once per day, five times per week) alone (osi), osimertinib intercalated every week with pemetrexed (100 mg/kg once a day, twice per week) (osi → pem), or osimertinib in combination with pemetrexed intercalated with osimertinib alone (osi + pem → osi). Tumor volume was measured twice per week and data are expressed as volume ± SEM (*n* = 8 tumors per group). *p < 0.05, **p < 0.01, ****p < 0.0001 vs osimertinib; two-way repeated measures analysis of variance followed by Bonferroni’s post-test. **b** Body weight was measured twice per week and data are expressed as percent change in body weight ± SEM (*n* = 8 tumors per group). *p < 0.05 vs osimertinib; two-way repeated measures analysis of variance followed by Bonferroni’s post-test. **c** The indicated treatments were stopped on day 110, and tumor growth was monitored for further 28 days in four animals per group. **d** Data are expressed as percent change in tumor volume: t corresponds to the tumor volume on day 138 (28 days after drug removal) and t_0_ corresponds to the tumor volume on day 110. **p < 0.01; Student’s t test was used for the comparison of means of the two groups

PC9T790M cells were subcutaneously inoculated into Balb/c-Nude female mice and after tumors had reached an average size of about 150mm^3^ the animals were randomized into four different groups: vehicle alone (ctrl); osimertinib (osi), osimertinib intercalated, every week, with pemetrexed (pem) (osi → pem); and osimertinib plus pemetrexed intercalated, every week, with osimertinib alone (osi + pem→osi). As illustrated in Fig. 1a, the continuous osimertinib treatment inhibited tumor growth up to 50 days. Subsequently, four of eight animals treated with osimertinib alone developed acquired resistance. When osimertinib was intercalated with pemetrexed (osi → pem) the inhibition of tumor growth was observed for 30 days, after which all the tumors started to grow again. Conversely, in the group of animals treated with osimertinib plus pemetrexed intercalated, every week, with osimertinib alone (osi + pem → osi), tumor regression was observed all along the experiment in all mice. Tumor growth was monitored for 110 days and during this period mice showed no signs of toxicity and regularly gained body weight (Fig. 1b).

In order to test the treatment efficacy in tumor eradication, osimertinib or osimertinib combined with pemetrexed treatments were stopped and regrowth of small tumors was monitored. All four small tumors from osimertinib monotherapy rapidly relapsed, while no relapses were observed when osimertinib had been co-administered with pemetrexed (Fig. 1c, d).

**Fig. 2:**
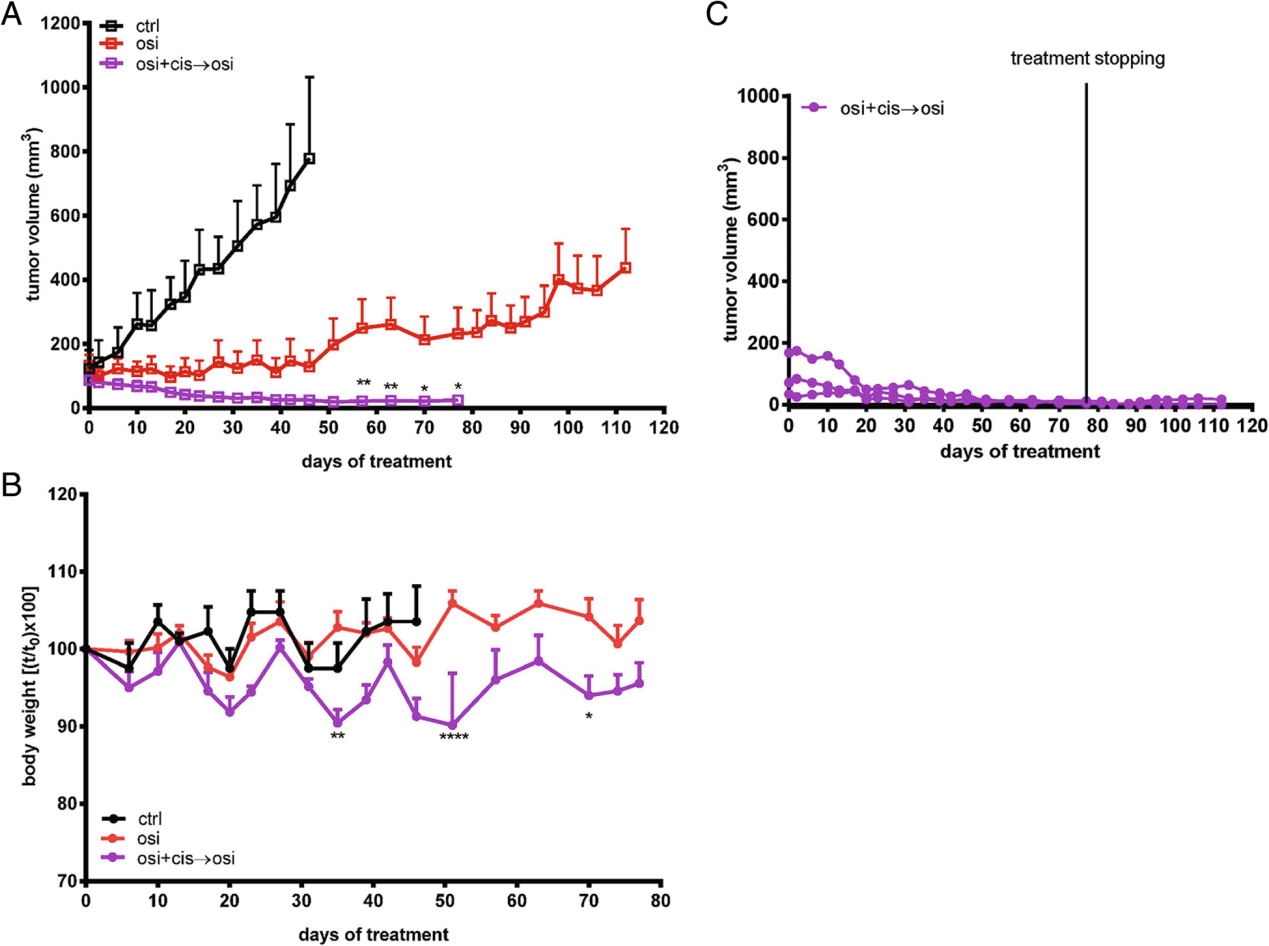
Effects of osimertinib and cisplatin combination in PC9T790M xenograft model. **a** PC9T790M cells were subcutaneously inoculated into BALB/C nude female mice, and after tumors had reached an average size of approximately 150mm^3^ the animals were treated with vehicle alone (ctrl), osimertinib (3 mg/kg once per day, five times per week) alone (osi), or osimertinib in combination with cisplatin (4 mg/kg once a day, twice per week) intercalated every week with osimertinib alone (osi + cis → osi). Tumor volume was measured twice per week and data are expressed as volume ± SEM (*n* = 8 tumors per group). *p < 0.05, **p < 0.01, vs osimertinib; two-way repeated measures analysis of variance followed by Bonferroni’s post-test. **b** Body weight was measured twice per week and data are expressed as percent change in body weight ± SEM (*n* = 8 tumors per group). *p < 0.05, **p < 0.01, ****p < 0.0001 vs osimertinib; two-way repeated measures analysis of variance followed by Bonferroni’s post-test. **c** The combined treatment was stopped on day 77, thereafter the tumor growth was monitored for further 35 days in four animals

Subsequently, we tested whether also cisplatin may exert a persistent tumor-inhibitory effect in PC9T790M xenografts when combined with osimertinib. PC9T790M cells were subcutaneously inoculated into Balb/c-Nude female mice and after tumors had reached an average size of about 150mm^3^ the animals were randomized into three different groups: vehicle alone (ctrl); osimertinib (osi); and osimertinib plus cisplatin (cis) intercalated, every week, with osimertinib alone (osi + cis → osi). As shown in Fig. 2a, osimertinib treatment inhibited tumor growth up to 45 days, then acquired resistance developed in four of the eight animals. In contrast, a significant inhibition of tumor growth was observed in all mice treated with osimertinib combined with cisplatin intercalated, every week, with osimertinib alone. However, differently from the combination with pemetrexed, mice treated with osimertinib plus cisplatin showed signs of toxicity (Fig. 2b) and treatment was stopped after 78 days. Interestingly, none of the animals treated with the drug combination showed a regrowth of tumors after treatment interruption (Fig. 2c).

**Fig. 3:**
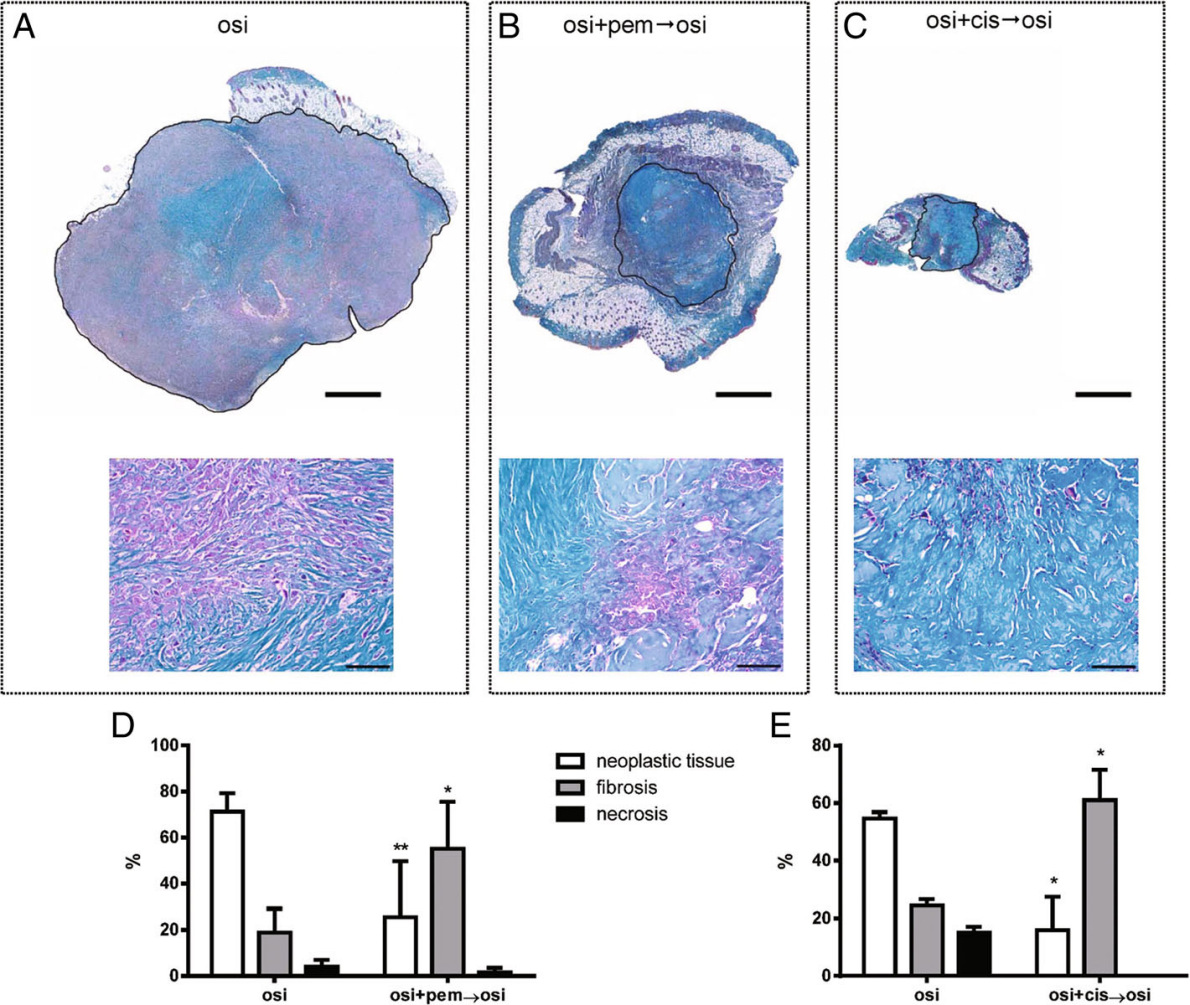
Morphometric analysis of tumor xenografts. Sections from PC9T790M xenografts treated with osi (**a**), osi + pem → osi (**b**) or osi + cis → osi (**c**) stained by Masson’s Trichrome to distinguish the fibrotic tissue (greenish) from neoplastic cells (purple). Tumors are encircled by black lines to exclude the skin, adnexa and uninvolved soft tissues. Scale bars = 1 mm. Higher magnifications of the same samples are shown on corresponding lower panels in which the increased amount of collagen deposition after treatment with osi + pem → osi or osi + cis → osi is apparent. Scale bars = 100 μm. Bar graphs showing the quantitative evaluation of tissue composition (neoplastic tissue, fibrosis and necrosis) in tumor xenografts following pharmacological treatment with osi alone or in association with pem (**d**) or cis (**e**). *p < 0.05, **p < 0.01 vs osi group; Student’s t-test

The quantitative evaluation of xenograft tissue composition was morphometrically determined to assess the effect of the different therapeutic schedules on the actual tumor mass, excluding fibrosis and necrosis (Fig. 3). Thus, on Masson’s trichrome stained sections, we documented that, compared to tumors from mice treated only with osimertinib, the combination of the drug with pemetrexed (Fig. 3d) or cisplatin (Fig. 3e) effectively reduced the neoplastic component of xenografts by 2.79 fold and 3.42 fold, respectively. The shrinkage in neoplastic area was coupled by a consensual increase in fibrotic tissue while necrosis did not significantly contributed to the effect of combinatory treatments.

Potential mechanisms of resistance to osimertinib were evaluated in all the tumors (eight) progressed to osimertinib. In one osimertinib-resistant tumor, *MET* was found amplified by FISH analysis and a higher expression level of MET was also confirmed by western blot analysis, suggesting *MET* amplification as a potential mechanism of resistance to osimertinib (Additional file 1: Figure S1). In the other resistant tumors (seven), analysis performed with NGS did not reveal any of the main acquired mechanisms of resistance described in the literature [5, 7]. Tertiary acquired mutations in *EGFR* gene were also excluded with ddPCR, performed to avoid possible NGS false-negative results due to sensitivity limit. In all the resistant tumors the *EGFR* setting of parental PC9T790M clone (p.(Glu746_Ala750del) and p.T790M) was maintained. In six resistant tumors, variants in *FGFR3, FOXL2, GNAQ* and *H3F3A*, which were not present in the parental PC9T790M clone, were identified (Additional file 2: Table S1).

The effects of osimertinib combined with pemetrexed or cisplatin on cell proliferation and cell death in PC9T790M cells in in vitro experiments were concomitantly evaluated.

**Fig. 4:**
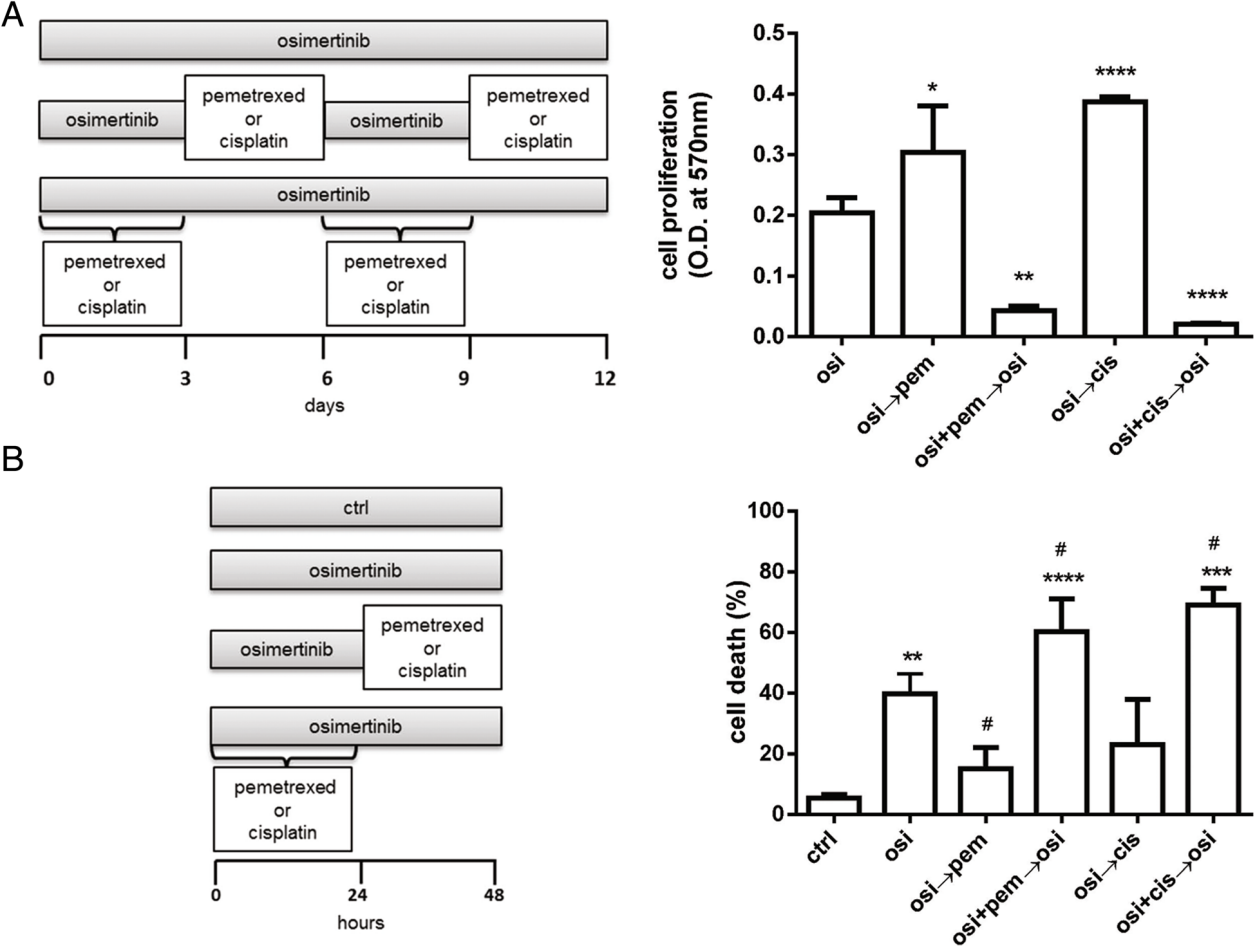
Effects of osimertinib combined with pemetrexed or cisplatin on cell proliferation and cell death induction in PC9T790M cells. **a** PC9T790M cells were treated with 50 nM osimertinib, 50 nM pemetrexed or 500 nM cisplatin on the basis of the indicated schedules. After 12 days crystal violet assay was performed and absorbance was measured at 570 nm. Results are representative of three independent experiments. *p < 0.05, **p < 0.01,***p < 0.001, ****p < 0.0001 vs osimertinib; one-way analysis of variance followed by Bonferroni’s post-test. **b** PC9T790M cells were treated with drugs on the basis of the indicated schedules for up to 48 h. At the end of the treatment period cell death was quantified by fluorescence microscopy analysis on Hoechst 33342 and propidium iodide-stained cells. Results represent the mean ± standard deviation (SD) of three independent experiments. **p < 0.01, ***p < 0.001, ****p < 0.001 vs control; #*p* < 0.05 vs osimertinib; one-way analysis of variance followed by Bonferroni’s post-test

As previously reported for gefitinib and pemetrexed [10], also for osimertinib the schedule of treatment in which osimertinib was given before pemetrexed or cisplatin was the less effective in inhibiting cell proliferation in long-term experiments and exerted an antagonist effect when alternated with pemetrexed (Fig. 4a). By contrast, the simultaneous treatment with osimertinib and pemetrexed or cisplatin significantly suppressed cell growth (Fig. 4a) and, as shown in Fig. 4b, enhanced cell death up to 60% in short-term experiments.

### Effects of pemetrexed or cisplatin on PC9T790M osimertinib resistant clones

**Fig. 5:**
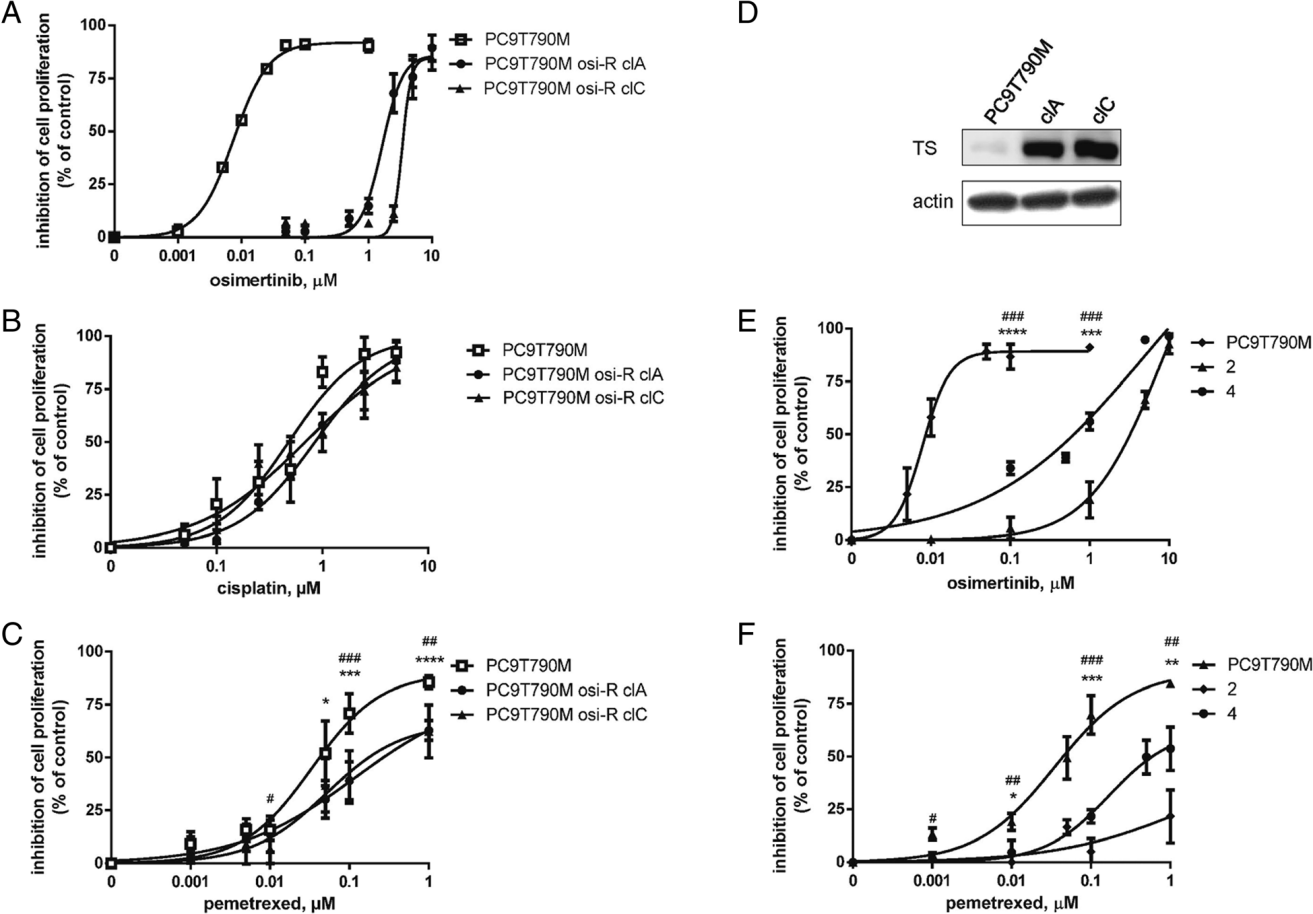
Effects of pemetrexed or cisplatin on PC9T790M osimertinib-resistant clones or on cell lines derived from osimertinib-resistant tumors. The osimertinib-resistant clones generated from PC9T790M (osi-R clA and osi-R clC) and the parental cells were treated with increasing concentrations of osimertinib (**a**), cisplatin (**b**) or pemetrexed (**c**). After 72 h cell proliferation was assessed by MTT assay. Results represent the mean ± standard deviation (SD) of at least three independent experiments. *p < 0.05, ***p < 0.001, ****p < 0.0001 vs osi-R clA; #*p* < 0.05, ##*p* < 0.01, ###*p* < 0.001 vs osi-R clC; Student’s t-test. **d** Thymidylate Synthase (TS) protein expression in PC9T790M, PC9T790M osi-R clA (clA), PC9T790 M osi-R clC (clC) cells evaluated by western blot analysis. Representative blot of two independent experiments is shown. PC9T790M cells and cells isolated from two osimertinib-resistant tumors (2 and 4) were treated with increasing concentrations of osimertinib (**e**) or pemetrexed (**f**). After 72 h cell proliferation was assessed by MTT assay. Results represent the mean (± standard deviation SD) of two independent experiments. *p < 0.05, **p < 0.01, ***p < 0.001, ****p < 0.001 vs 2; #*p* < 0.05, ##*p* < 0.01, ###*p* < 0.001 vs 4; Student’s t-test)

With the aim to test the chemosensitivity of osimertinib-resistant cells, we generated two clones (osi-R clA, clC) after 9 months of culturing PC9T790M cells in the presence of increasing concentrations of osimertinib (from 10 nM to 500 nM). Dose response treatment curves showed an IC_50_ for osimertinib higher than 5 μM (Fig. 5a). Both clones maintained the exon 19 deletion and T790M mutation in *EGFR,* but neither C797S, nor amplification of *MET* or *HER-2*, nor phenotypic transformation were identified as potential mechanisms of resistance (not shown). NGS analysis did not reveal any acquired mutations. Analysis of Copy Number Alteration (CNA) showed that osi-R clC presented an amplification of *NRAS*. This alteration was also confirmed by ddPCR CNA assay (Additional file 3: Figure S2).

Interestingly, when osimertinib was combined with pemetrexed or cisplatin no resistant clones emerged confirming results described in Fig. 4a.

As shown in Fig. 5b, c, the resistant clones were as sensitive to cisplatin as PC9T790M parental cells, but were more resistance to pemetrexed, as we previously reported also for PC9 cells resistant to gefitinib [10]. *TS* gene expression was significantly increased in clA and clC relative to parental cells (Fig. 5d), suggesting that this mechanism may be responsible for resistance, in keeping with published literature demonstrating that TS overexpression is a common mechanism for resistance to pemetrexed therapy in NSCLC [16]. However, we cannot rule out that other mechanisms involved in the resistance to osimertinib may also confer resistance to pemetrexed. To further confirm pemetrexed resistance in osimertinib-resistant cells, we established two cell clones from resistant tumors emerged in the osimertinib group of the experiment shown in Fig. 2a and, as expected, both cell clones showed an IC_50_ value for osimertinib greater than 5 μM (Fig. 5e). Accordingly with the results obtained with the resistant clones generated in vitro, also the cells derived from resistant tumors were more resistant to pemetrexed (Fig. 5f).

### Effects of osimertinib combined with pemetrexed or cisplatin on cell proliferation and cell death induction in PC9 and HCC827 cell lines

Considering that FDA and EMA approved osimertinib for first line treatment in EGFR mutated patients according to the results of FLAURA trial [4], we planned in vitro experiments on PC9 and HCC827 cell lines carrying *EGFR* mutations and sensitive to first-generation EGFR TKIs such as gefitinib and erlotinib.

**Fig. 6:**
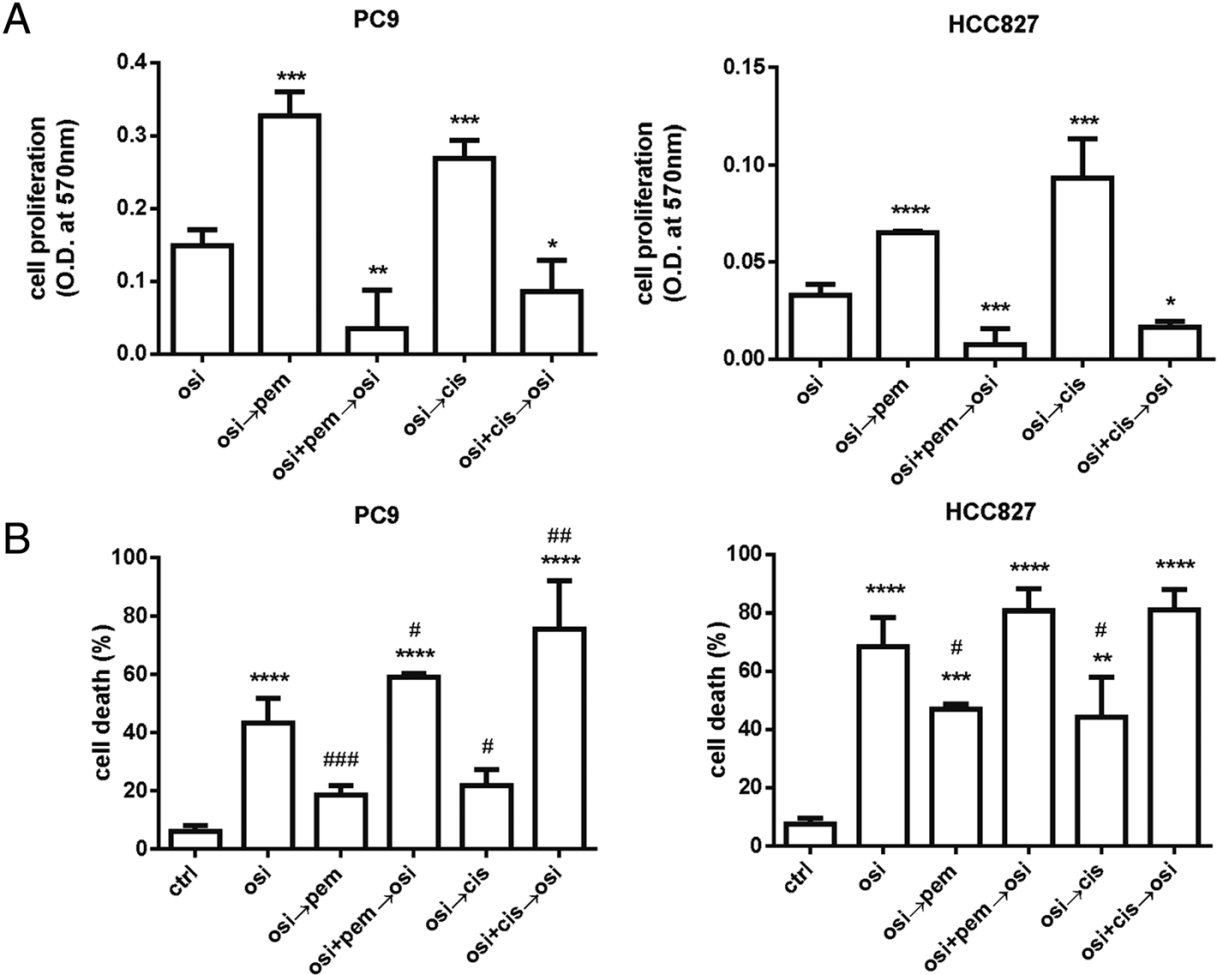
Effect of osimertinib combined with pemetrexed or cisplatin on cell proliferation and cell death induction in PC9 and HCC827 cell line. **a** PC9 and HCC827 cells were treated with 50 nM osimertinib, 50 nM pemetrexed or 500 nM cisplatin on the basis of schedules indicated in Fig. 4a. After 12 days crystal violet assay was performed and absorbance was measured at 570 nm. Results are representative of three independent experiments. *p < 0.05, **p < 0.01, ***p < 0.001, ****p < 0.0001 vs osimertinib; one-way analysis of variance followed by Bonferroni’s post-test. **b** PC9 and HCC827 cells were treated with 50 nM osimertinib, 50 nM pemetrexed or 500 nM cisplatin on the basis of the schedules indicated in Fig. 4b. At the end of the treatment period cell death was quantified by fluorescence microscopy analysis on Hoechst 33342 and propidium iodide-stained cells. Results represent the mean (± standard deviation SD) of three independent experiments. ****p < 0.001 vs control; #*p* < 0.05, ##*p* < 0.01, ###*p* < 0.001 vs osimertinib; one-way analysis of variance followed by Bonferroni’s post-test

Results obtained in term of inhibition of cell proliferation and induction of cell death when the cells were exposed to different treatments of osimertinib combined with pemetrexed or cisplatin (Fig. 6) were comparable to those observed for PC9T790M cells and previously described (see Fig. 4 for details). These data, even if not confirmed in xenograft models, suggest that also for naïve patients the presence of pemetrexed or cisplatin may enhance the efficacy of osimertinib in first-line treatment.

**Fig. 7:**
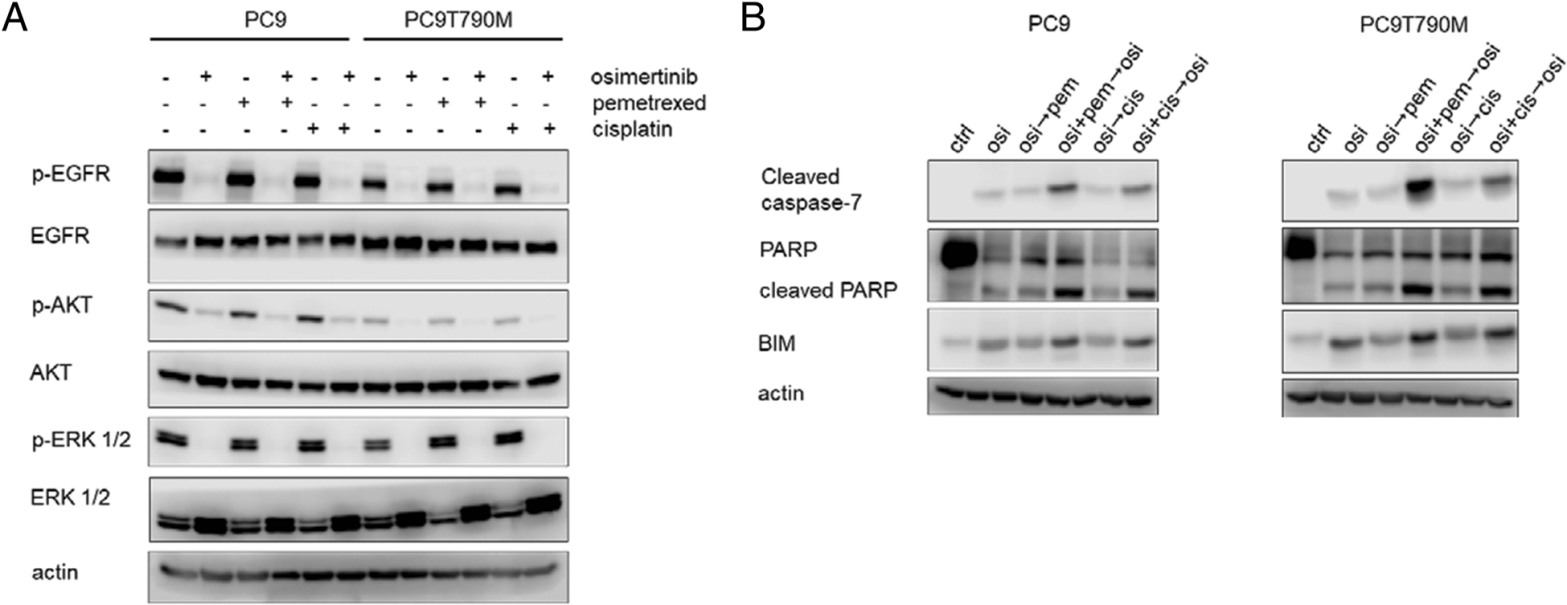
Effect of osimertinib combined with pemetrexed or cisplatin on signaling transduction pathways and cell death regulators in PC9 and PC9T790M cell lines. PC9 and PC9T790M cells were treated with 50 nM osimertinib, 50 nM pemetrexed, 500 nM cisplatin or their combination and after 24 h (**a**) or 48 h following the schedules indicated in Fig. 4b (**b**) the cells were lysed and western blot analysis was performed to detect the indicated proteins. Results are representative of two independent experiments

We then studied the effect of the association of osimertinib with pemetrexed or cisplatin on intracellular modulation of transduction pathways and expression of cell death regulators (Fig. 7). EGFR, AKT and ERK activation (Fig. 7a) were strongly inhibited by osimertinib both in PC9 and in PC9T790M cells as expected, being these cell lines highly responsive to osimertinib. Pemetrexed or cisplatin did not affect the phosphorylation status of these proteins and the combination with osimertinib did not differ from osimertinib alone.

By contrast, the combined treatment strongly induced the proapoptotic BCL-2 family member Bim, enhanced the activation of caspase 7 and the cleavage of PARP (Fig. 7b).

## Discussion

Although preclinical and clinical researches have explored the interaction of first-generation EGFR-TKIs and cytotoxic agents [10, 17–23], to date there are no data on preclinical combination of chemotherapy with third-generation EGFR-TKIs, such as osimertinib. The tolerability of osimertinib monotherapy indeed may allow for the development of more efficacious combination regimens.

In this study, we explored the efficacy of osimertinib combined with pemetrexed or cisplatin in NSCLC PC9T790M nude mice xenografts. A strong anti-tumor effect was observed when osimertinib was combined with pemetrexed or cisplatin intercalated, every week, with osimertinib alone. Following this approach, no tumor became resistant, differently from the treatment with osimertinib alone which induced acquired resistance in 50% of mice. Very interestingly, the combination treatment enhanced the percentage of fibrotic tissue within the xenograft tumors and the small tumors did not regrow when the administration of drugs was stopped, indicating a stronger efficacy in eradicating parenchymal tumor cells.

A combination of cetuximab and trastuzumab with osimertinib has been very recently reported as a new strategy for preventing resistance to osimertinib with strong and durable effects in animal studies [24, 25].

Regarding first generation EGFR TKIs, in two recent phase II randomized trials [21, 22], patients with treatment-naive advanced EGFR mutant NSCLC were treated with continuous gefitinib in combination with chemotherapy (pemetrexed in [22] and carboplatin-pemetrexed in [21]). In both trials, patients on the combination with continuous gefitinib and chemotherapy experienced a statistically significant longer PFS than gefitinib alone (16.2 months versus 11.1 months in [22]; 17.5 months versus 11.9 months in [21]).

Another randomized trial evaluating a concurrent vs a sequential regimen of gefitinib and carboplatin-pemetrexed in first-line [23] demonstrated a PFS and OS benefit when gefitinib was given concomitantly with chemotherapy. The sequential regimen in which gefitinib was given before chemotherapy, a schedule that in our and other preclinical studies had shown antagonistic effect [10, 19, 20], demonstrated a worse outcome.

The concurrent regimen is currently being evaluated against gefitinib alone in a randomized phase III study recently presented at ESMO 2018 meeting [26]. In this trial, the patients who received a combination of gefitinib with carboplatin-pemetrexed showed a statistically significant benefit in survival (PFS of 20.9 vs 11.2 months, *p* < 0.001 and OS of 52.2 vs 38.8, *p* = 0.013 for gefitinib and carboplatin/pemetrexed and for gefitinib alone, respectively).

Our results indicate that, osimertinib given before pemetrexed was the worst therapy to delay resistance. By contrast, the combination of osimertinib with pemetrexed or cisplatin prevented or at least delayed the acquisition of resistance and inhibited regrowth when treatment was stopped with tumor cure. We did not observe tumor cures in the osimertinib monotherapy group, strongly indicating that the addition of chemotherapy may potentiate the efficacy of osimertinib either in term of inhibition of tumor growth or appearance of relapses.

In PC9 and PC9T790M cell lines, analysis of signaling transduction pathways and protein related to cell death revealed that the combination treatment did not affect the intracellular transduction pathways, which were already completely suppressed by osimertinib alone, but strongly enhanced apoptosis signaling via caspase-7 activation. This observation may be of relevance for the results obtained in vivo.

*EGFR* mutation-positive NSCLC is highly heterogeneous at the cellular level; therefore, the selective pressure exerted by TKIs may promote the clonal expansion of resistant clones through different molecular mechanisms [27, 28]. Regardless of the resistance mechanisms that eventually will develop, it is conceivable that the enhancement in cell death associated with osimertinib/chemotherapy combination, presumably triggered also in vivo, may postpone the emergence of resistance to EGFR-TKIs by limiting the original tumor heterogeneity. Then, a delay in the clonal expansion of pre-existing resistant clones may reasonably explain the results from in vivo experiments. However, it is worth noting that chemotherapy itself exerts a selective pressure that may influence tumor heterogeneity and subsequent clonal evolution. Therefore, despite we cannot conclude that the combined approach could completely prevent the resistance, in the timing we performed the experiments no resistant tumors developed when mice were treated with osimertinib combined with pemetrexed or cisplatin.

For patients with T790M positive tumors progressing after second-line osimertinib, chemotherapy remains the standard of care. However, our data indicate that osimertinib resistant clones, obtained after 9 months of selection with increasing concentrations of the drug or isolated from osimertinib resistant xenografts, were less sensitive to pemetrexed with respect to parental cells. These clones showed a marked increase in TS expression, which reduces the efficacy of pemetrexed [16].

Differently, the responsiveness to cisplatin remained unchanged. These results suggest to use a chemotherapy regimen not-containing pemetrexed in patients progressing after osimertinib; no clinical data are available about the best chemotherapy regimen in this setting.

Potential mechanisms of resistance were evaluated in all the tumors progressed to treatment and in resistant cell lines. Apart from *MET* amplification, detected in one tumor, we have not been able to identify other already known mechanisms responsible for the lack of responsiveness to osimertinib. Nevertheless, in resistant tumors we reported (see Additional files) SNV and/or CNA detected by NGS analysis, but actually little is known about their involvement in mechanisms of acquired resistance to osimertinib and this aspect remains a matter for further investigation. *FGFR3*, observed in one tumor, was described only as a partner in oncogenic fusion, no missense variants have been reported until now as mediators of resistance [29]. *NRAS* amplification, observed in osi-R clC, could potentially be a mechanism of resistance to osimertinib. Indeed, this CNA was observed as mechanism of resistance in vitro to naquotinib, a third-generation EGFR inhibitor [30]. *FOXL2, GNAQ* and *H3F3A* have not been reported in the literature as associated with acquired resistance to osimertinib, and these variants will undergo functional characterization to elucidate their potential role.

In view of the superior efficacy of osimertinib in the first-line treatment [4], we tested in vitro, in PC9 and HCC827 cell lines, the combination between osimertinib and pemetrexed or cisplatin. The advantage in using the combinatory regimen was demonstrated also in these cell lines harbouring* EGFR* activating mutation without T790M, suggesting that cytotoxic agents should be added to EGFR-TKIs as first-line therapy in advanced NSCLC with activating *EGFR *mutation.

## Conclusion

Our preclinical results provide a strong rationale for randomized studies comparing osimertinib vs osimertinib plus chemotherapy, either in* EGFR* T790M positive and also in EGFR-TKI naïve NSCLC patients. In particular, a phase III trial evaluating osimertinib combined with platinum-pemetrexed vs. osimertinib alone could be the right step forward to significantly prolong the survival of *EGFR-*mutated NSCLC patients.

## Additional files

Additional file 1: **Figure S1.** MET analysis in a tumor progressed to osimertinib. (TIF 873 kb)

Additional file 2: **Table S1.** Analysis of putative mechanism of acquired resistance to osimertinib: Next Generation Sequencing results. (TIFF 13 kb)

Additional file 3: **Figure S2.** ddPCR assay of NRAS amplification in osimertinib-resistant clones generated from PC9T790M. (TIFF 1494 kb)

## Data Availability

Data and materials will be shared.

## Abbreviations

CNA: Copy Number Alteration
EGFR: Epidermal Growth Factor Receptor
NSCLC: Non-Small Cell Lung Cancer
PFS: Progression-Free Survival
SNV: Single Nucleotide Variation
TKIs: Tyrosine Kinase Inhibitors
TS: Thymidylate Synthase
