# Supplementary figures and images for: Third generation EGFR inhibitor osimertinib combined with pemetrexed or cisplatin exerts long-lasting anti-tumor effect in EGFR-mutated pre-clinical models of NSCLC
Source: J Exp Clin Cancer Res. 2019 May 28;38:222. doi: 10.1186/s13046-019-1240-x (PMC6537372; doi:10.1186/s13046-019-1240-x)

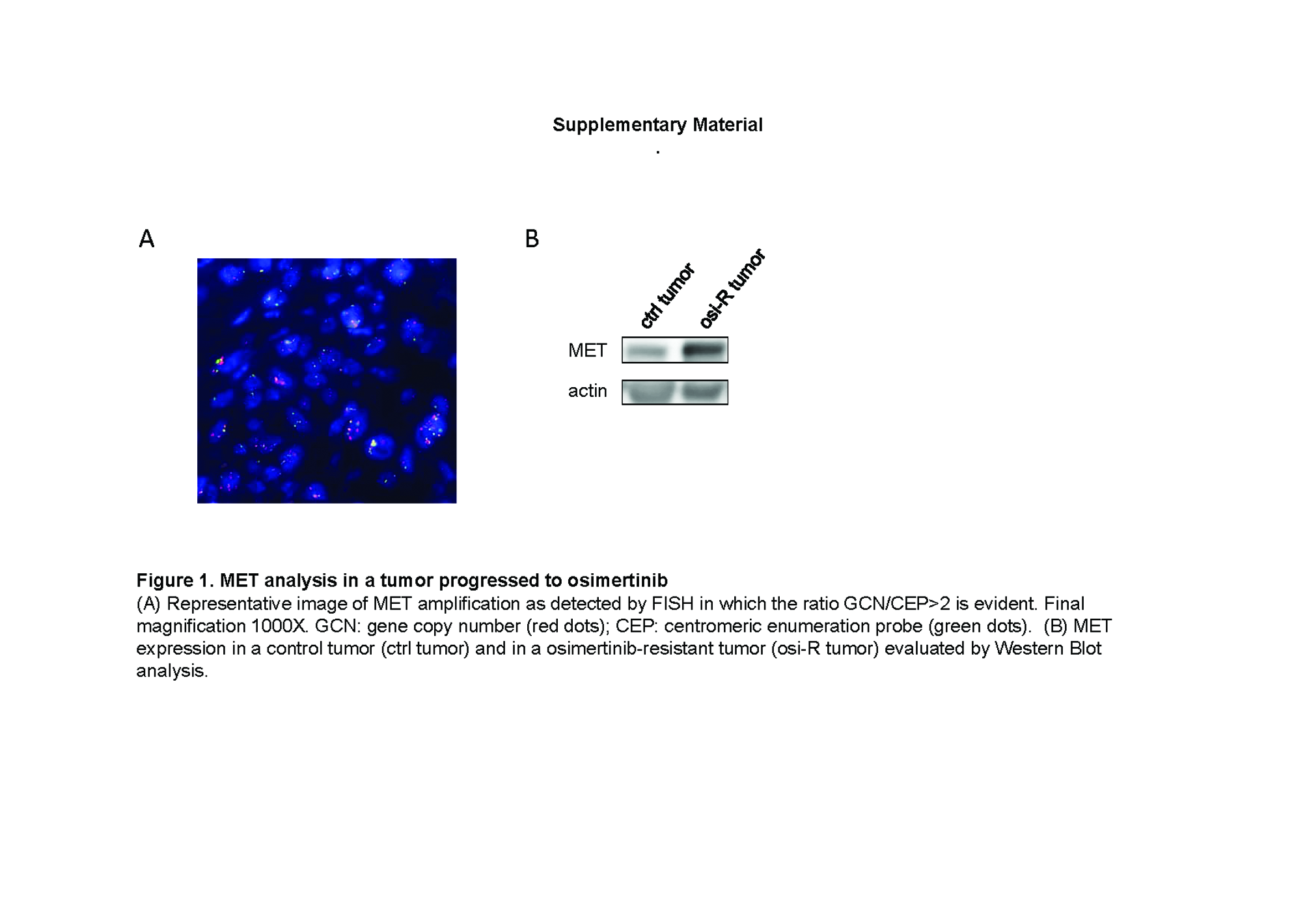

Supplement: Supplementary file 1 — Figure S1. MET analysis in a tumor progressed to osimertinib. (TIF 873 kb) [file 13046_2019_1240_MOESM1_ESM.tif]

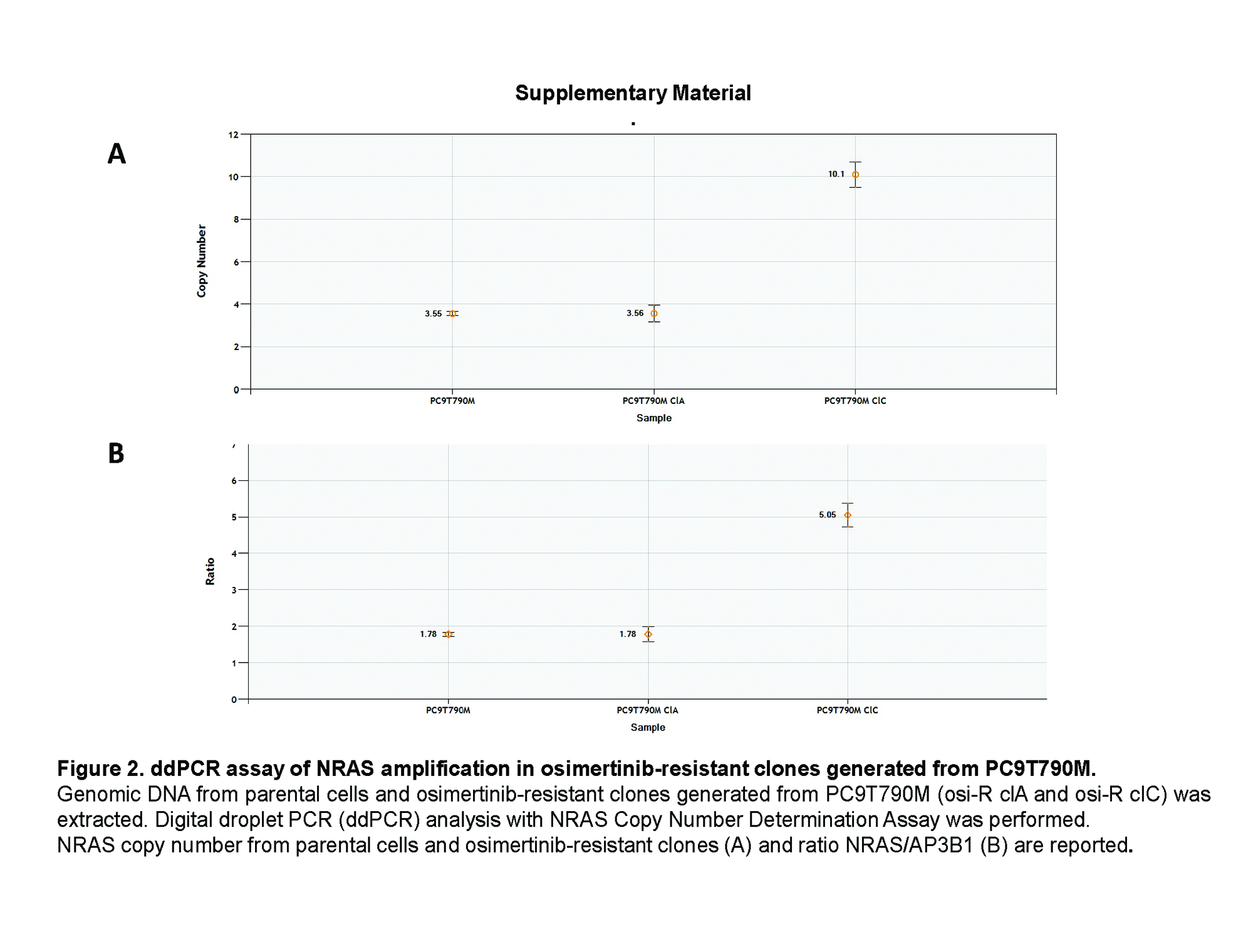

Supplement: Supplementary file 3 — Figure S2. ddPCR assay of NRAS amplification in osimertinib-resistant clones generated from PC9T790M. (TIFF 1494 kb) [file 13046_2019_1240_MOESM3_ESM.tiff]
